# Feature tracking (FT) and extracelluar volume (ECV) by cardiac magnetic resonance segmentally analyze change of LV in Ebstein:a novel perspective in myocardial remodeling

**DOI:** 10.1186/1532-429X-18-S1-O31

**Published:** 2016-01-27

**Authors:** Dan Yang, Jiayu Sun, Yong Luo, Hong Liu, Ke Wan, Tianjing Zhang, Yucheng Chen

**Affiliations:** 1 Cardiology Division, West China Hospita Sichuan University, Chengdu, China; 2 Radiology, West China Hospital Sichuan University, Chengdu, China; 3 Northeast Asia MR Collaboration, Siemens Healthcare, Beijing, China

## Background

Ebstein anomaly is classically considered a malformation of right heart, however, left ventricular is also affected. As LV heart failure is the most common during the natural history, an understanding of heart failure modes is quite significant. Some study have described that the Ebstein's LV basal narrowing, apical modestly dilation, and basal septal dyskinesis but in most ebstein patients the LVEF preserved. The mechanism of the change of LV of Ebstein is not conmon known, but its clinic value draw us searching deeper.

So we sought to assess the segmental change in cardiovascular magnetic resonance (CMR) tissue tracking parameters and segmentally quantify Ebstein's LV degree of myocardial diffuse fibrosis by extracelluar volume fraction (ECV), in turn explore the potential mechanism of the LV change

## Methods

**Figure 1:**
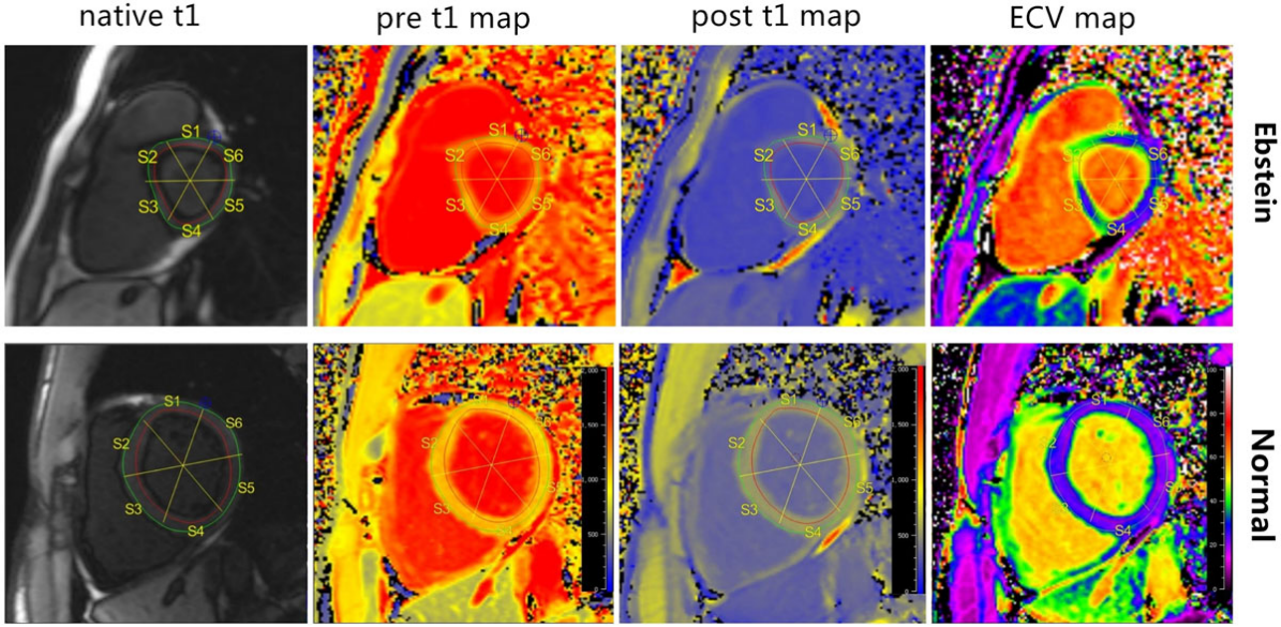
**The method that measuring the segmental ECV of the basal left ventricle listed as native t1, pre t1 map, post t1 map, ECV map**.

15 ebstein patients and 15 age- and sex- matched normal controls were included in current analysis. Three dimensional peak LV strain, peak systolic/circumferential strain-rate, time to peak radial/circumferential strain were measured using 2 chamber (2c), 4 chamber (4c) and stacks of short axis slices in diastolic phase, acquired 16 segments strain, strain rate and time to peak. Subsequently, we manually calculated LV septal/free wall strain and SD time to peak, maximum wall delay and AS-IL time to peak. Segmental ECV were measured on short axis on basal-and mid-ventricular which were corresponding to tissue tracking slices (figure 1). Paired t-test was performed to compare the difference between segments of strain and ECV between Ebstein patients and controls.

## Results

**Figure 2:**
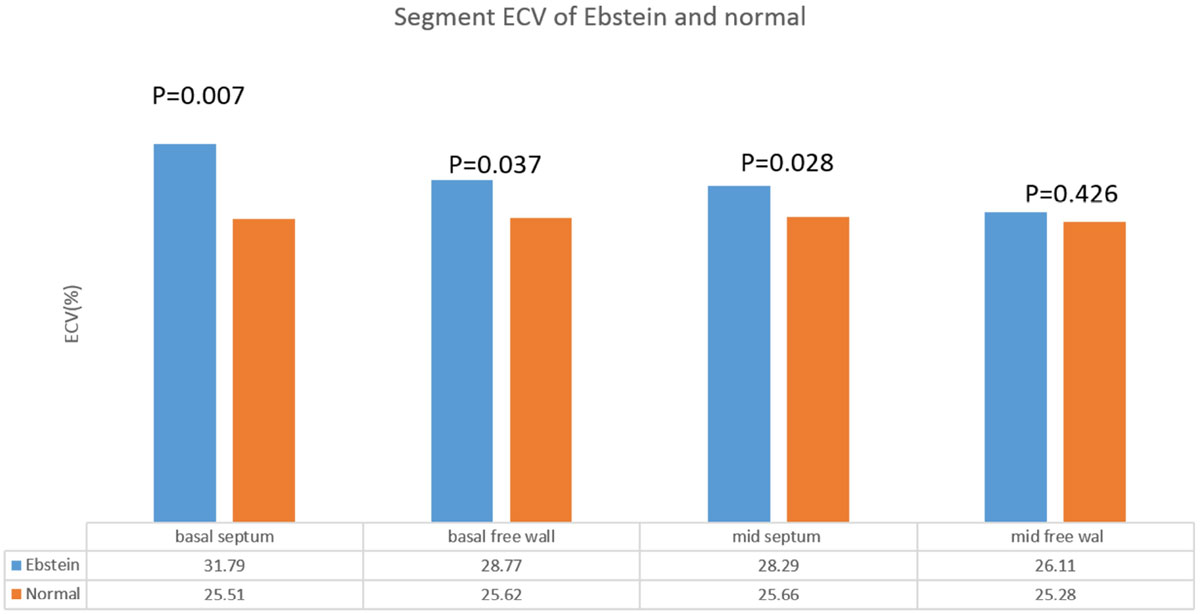
**The segmental ECV of basal and mid ventricle of ebstein patients and controls, and below excel is ECV value listed as mean**.

No difference was found between patients and controls at age (29.85 years vs.29.35 years p = 0.769). The radial and circumferential strain were increasing from basal to apical slices in both patients and controls (P < 0.05). Basal septal radial and circumferential strain in Ebstein decreased comparing with controls(radial 14.61 ± 7.38 vs 24.38 ± 3.37 p < 0.001, circumferential -3.54 ± 11.23 vs -15.63 ± 1.64 P = 0.001), but surprisingly, mid and apical septal radial/circumferential strain were larger in Ebstein. Correspondingly, the basal septal ECV were higher than other segmental ECV in patients and all segments in controls (figure 2). The whole LV motion delayed in patients comparing to normal controls(P < 0.05), especially the basal septum (radial:SD time to peak 81.21(35.35,208.7) vs 48.82(29.69,91.79) p = 0.015, Maximum wall delay:204.62(79.92,475.44) vs 124.57(60.4,250.88) p = 0.017 AS-IL time to peak:71.38(-147,441.48) vs -48.066(-100.08,61.36) p = 0.016)).

## Conclusions

FT and ECV are sensitive to document segmental change in cardiac function and histopathology. Additionally, FT and ECV together illustrated that diffuse myocardial fibrosis also existed differential distribution in patients, which negatively related to segmental change in cardiac function. This would be a novel perspective in clinically therapy and prognosis assessment.

